# Inhibition of the CXCL12/CXCR4-Axis as Preventive Therapy for Radiation-Induced Pulmonary Fibrosis

**DOI:** 10.1371/journal.pone.0079768

**Published:** 2013-11-07

**Authors:** Hui-Kuo G. Shu, Younghyoun Yoon, Samuel Hong, Kaiming Xu, Huiying Gao, Chunhai Hao, Edilson Torres-Gonzalez, Cardenes Nayra, Mauricio Rojas, Hyunsuk Shim

**Affiliations:** 1 Department of Radiation Oncology, Emory University School of Medicine, Atlanta, Georgia, United States of America; 2 Winship Cancer Institute, Emory University School of Medicine, Atlanta, Georgia, United States of America; 3 Department of Radiology and Imaging Sciences, Emory University School of Medicine, Atlanta, Georgia, United States of America; 4 Department of Pathology, Emory University School of Medicine, Atlanta, Georgia, United States of America; 5 Department of Medicine, University of Pittsburgh, Pittsburgh, Pennsylvania, United States of America; Children's Hospital Los Angeles, United States of America

## Abstract

**Background:**

A devastating late injury caused by radiation is pulmonary fibrosis. This risk may limit the volume of irradiation and compromise potentially curative therapy. Therefore, development of a therapy to prevent this toxicity can be of great benefit for this patient population. Activation of the chemokine receptor CXCR4 by its ligand stromal cell-derived factor 1 (SDF-1/CXCL12) may be important in the development of radiation-induced pulmonary fibrosis. Here, we tested whether MSX-122, a novel small molecule and partial CXCR4 antagonist, can block development of this fibrotic process.

**Methodology/Principal Findings:**

The radiation-induced lung fibrosis model used was C57BL/6 mice irradiated to the entire thorax or right hemithorax to 20 Gy. Our parabiotic model involved joining a transgenic C57BL/6 mouse expressing GFP with a wild-type mouse that was subsequently irradiated to assess for migration of GFP+ bone marrow-derived progenitor cells to the irradiated lung. CXCL12 levels in the bronchoalveolar lavage fluid (BALF) and serum after irradiation were determined by ELISA. CXCR4 and CXCL12 mRNA in the irradiated lung was determined by RNase protection assay. Irradiated mice were treated daily with AMD3100, an established CXCR4 antagonist; MSX-122; and their corresponding vehicles to determine impact of drug treatment on fibrosis development. Fibrosis was assessed by serial CTs and histology. After irradiation, CXCL12 levels increased in BALF and serum with a corresponding rise in CXCR4 mRNA within irradiated lungs consistent with recruitment of a CXCR4+ cell population. Using our parabiotic model, we demonstrated recruitment of CXCR4+ bone marrow-derived mesenchymal stem cells, identified based on marker expression, to irradiated lungs. Finally, irradiated mice that received MSX-122 had significant reductions in development of pulmonary fibrosis while AMD3100 did not significantly suppress this fibrotic process.

**Conclusions/Significance:**

CXCR4 inhibition by drugs such as MSX-122 may alleviate potential radiation-induced lung injury, presenting future therapeutic opportunities for patients requiring chest irradiation.

## Introduction

Cancer therapy may require radiation treatment in the chest, potentially resulting in significant dose to lung tissue. These patients are at risk for developing lung radiation injury including pulmonary fibrosis (PF), an incurable, late radiation toxicity that can cause significant morbidity and even mortality depending on the volume of lung affected (For review, see [1]). After radiation exposure, Type I pneumocytes are depleted with accompanying Type II pneumocyte hyperplasia as part of the process of alveolar epithelial regeneration. During this process, local cytokine/chemokine production leads to recruitment and retention of inflammatory cells including macrophages. As acute inflammation resolves, fibroblasts are recruited, resulting in interstitial collagen deposition and alveolar septal thickening. 

Recruitment of fibroblasts is critical in the development of idiopathic PF (For review, see [2]). It is now known that bone marrow (BM)-derived fibroblast progenitor cells, known as fibrocytes, are recruited and likely play a major role in the fibrotic process [3]. Similarly, after bleomycin-induced injury, BM-derived fibrocytes, which express CXCR4, are recruited to fibrogenic regions of lung [4,5]. Neutralizing antibody against CXCL12 can prevent the recruitment of circulating fibrocytes to bleomycin-damaged lung and suppress the development of fibrosis [4]. Further characterization of these BM-derived cells demonstrate expression of the mesenchymal stem cell markers CD44 and CD105 in addition to CXCR4 but lack of the hematopoetic stem cell marker CD45 [5]. Therefore, the CXCR4/CXCL12-axis appears critical in recruiting BM-derived precursors that differentiate into the fibroblasts that cause PF. 

To date, several CXCR4 antagonists have been developed (For review, see [6]). TN14003, a 14-mer peptide, blocks development of PF in bleomycin-treated C57BL/6 mice [5]. AMD3100 (Plerixafor), an FDA-approved small molecule CXCR4 antagonist, has also been tested on bleomycin-treated mice. While AMD3100 is effective at blocking stem cell homing, it also increases stem cell mobilization, which has led to its use for increasing stem cell yields in preparation for autotransplantation. Consistent with its ability to block homing, Watanabe et al. found that initiating the drug prior to bleomycin exposure decreased development of PF and improved mouse survival [7]. However, if AMD3100 was initiated after bleomycin exposure, fibrosis actually increased possibly due, in part, to mobilization of stem cells from the bone marrow. 

While bleomycin- and radiation-induced PF has widely disparate latencies in mouse models ranging from 2-8 weeks (for bleomycin) to upwards of 6 months or more (for radiation) [8,9], they have similar mechanisms of action, namely production of DNA strand breaks. In addition, a genetic factor thought to predict susceptibility to bleomycin-induced PF similarly predicts radiation-induced PF [8]. 

In this study, we sought to characterize the CXCR4/CXCL12-axis in a mouse model of radiation-induced PF and assess the effect of blocking CXCR4 on the pathogenesis of this late toxicity. We recently reported the development of a unique partial anti-CXCR4 compound, MSX-122, that blocks homing of metastatic and inflammatory cells without mobilizing stem cells [10]. This drug can intervene in the Gα_i_-signaling pathway (cAMP modulation), but not the Gq-pathway (calcium flux). More details about differences between this partial anti-CXCR4 compound and previous inhibitors has been reported [10]. Here, we assessed the effectiveness of MSX-122 and AMD3100 at inhibiting radiation-induced PF. 

## Materials and Methods

### Species used and handling of animals

Female C57BL/6 mice were used for all experiments. All animals were housed in the Emory animal facility. Protocols for animal studies were reviewed and approved by the Institutional Animal Care and Use Committee at Emory University (IACUC protocol numbers:170-2008 and 065-2006). Emory DAR (Division of Animal Resources) has experienced clinical veterinary and animal caretaker support staff who are well-trained in the care of rodents. All animals were monitored daily by the investigators and/or by members of the clinical veterinary staff concerning general health and to detect signs of discomfort. Any animal showing signs of discomfort, pain or distress was sacrificed immediately following IACUC guideline for endpoints. All reasonable measures were taken to ensure the health and well being of the animals during the course of the study. The procedures to be used for animal care were consistent with those established by the NIH and the vivarium was fully accredited by the AAALAC. All experimental results adhere to the ARRIVE guidelines for the reporting of animal research [11]. 

### Thoracic irradiation models

**Figure 1:**
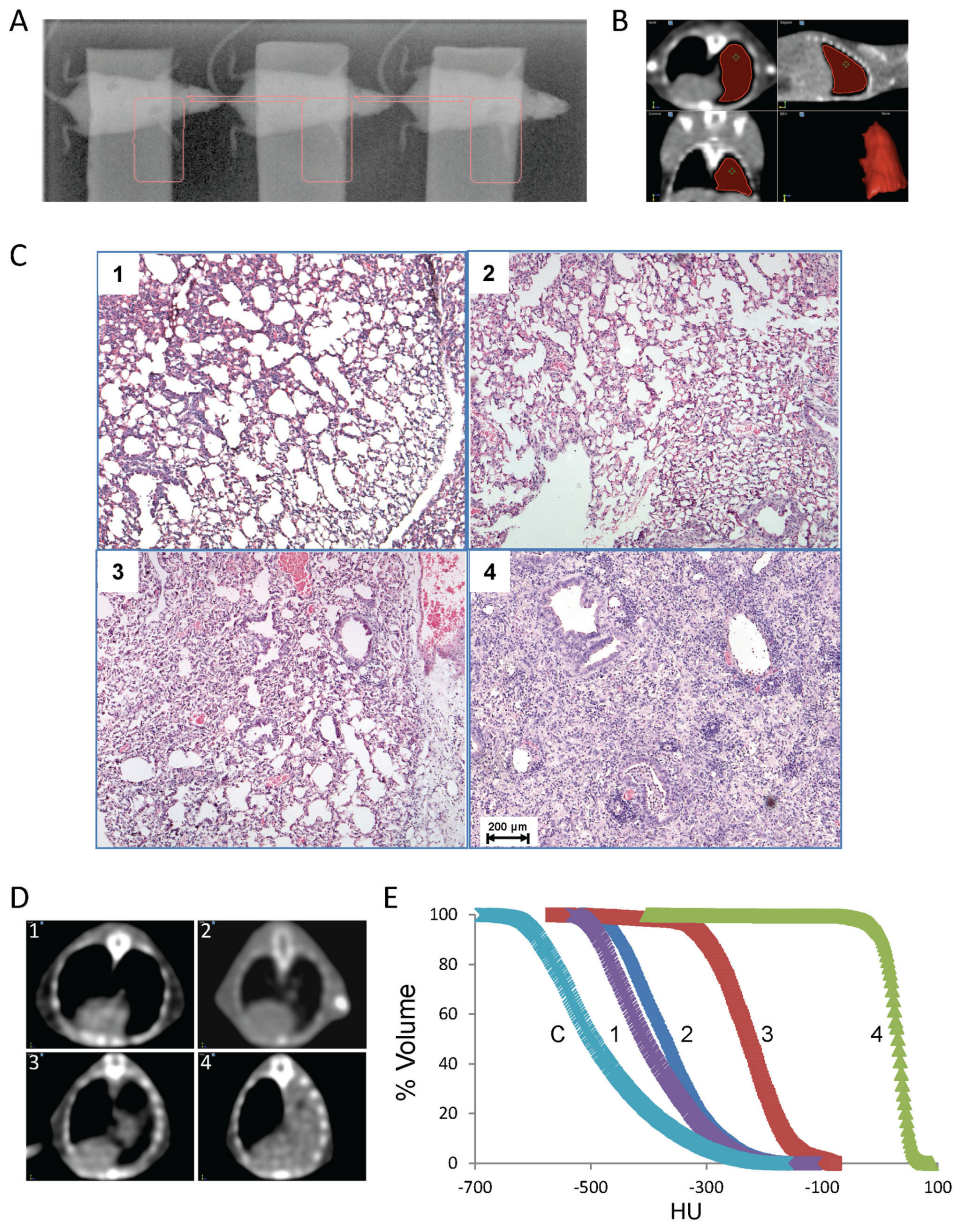
Quantification of PF in a hemithoracic irradiation model. *A*, Representative portal image of three mice receiving right hemithoracic radiation (region of treatment in pink) is shown. *B*, VelocityAI interface was used to show representative axial/coronal/sagittal slices of reconstructed CT image set with the right lung as 3D-volume of interest contoured (red). *C*-*E*, Representative H&E-stained lung slides (*C*), corresponding axial CT images (*D*) and plots of HU versus %volume (*E*) at C (untreated), 8(1), 12(2), 16(3), and 20(4) weeks after irradiation are shown.

All irradiation was performed on a clinical linear accelerator. For right hemithoracic irradiation, three 8-week old female C57BL/6 mice were anesthetized and thoracic regions corresponding to right lungs were aligned. 1.0 cm thickness water-density flexible bolus pieces were placed on top of the mice for dose buildup and collimated fields that encompassed the right hemithorax were used (Figure **1A**). For whole thoracic irradiation, 6 mice were simultaneously treated. 6 MV photons were used to deliver 20 Gy prescribed at Dmax (1.0 cm bolus + ~0.5 cm chest). All animal experiments were approved by the IACUC at Emory University. 

### CT image analysis

For initial testing, computed tomography (CT) scans were obtained on a Lightspeed RT-16 unit (GE Healthcare) with the following parameters: 120-kV, 100-mA, 9.6-cm FOV, 0.625-mm slice thickness over 3.5-cm scan region (entire lung). A 20 week post-irradiation CT scan was done for all drug- or vehicle-treated groups. CT images were then transferred to the VelocityAI software (Velocity Medical Systems), and a three-dimensional contour of the irradiated lung was determined (Figure **1B**). Plots of Hounsfield-units (HU) versus percentage of the irradiated lung volume (% volume) were generated. % volume > -200 HU was utilized as a marker for the extent of fibrosis. Also, CT- and histology-based assessments were normalized to percent-scale to evaluate the efficacy of the CXCR4 antagonists in attenuating radiation-induced PF. 

### Parabiotic model and flow cytometry analysis

**Figure 2:**
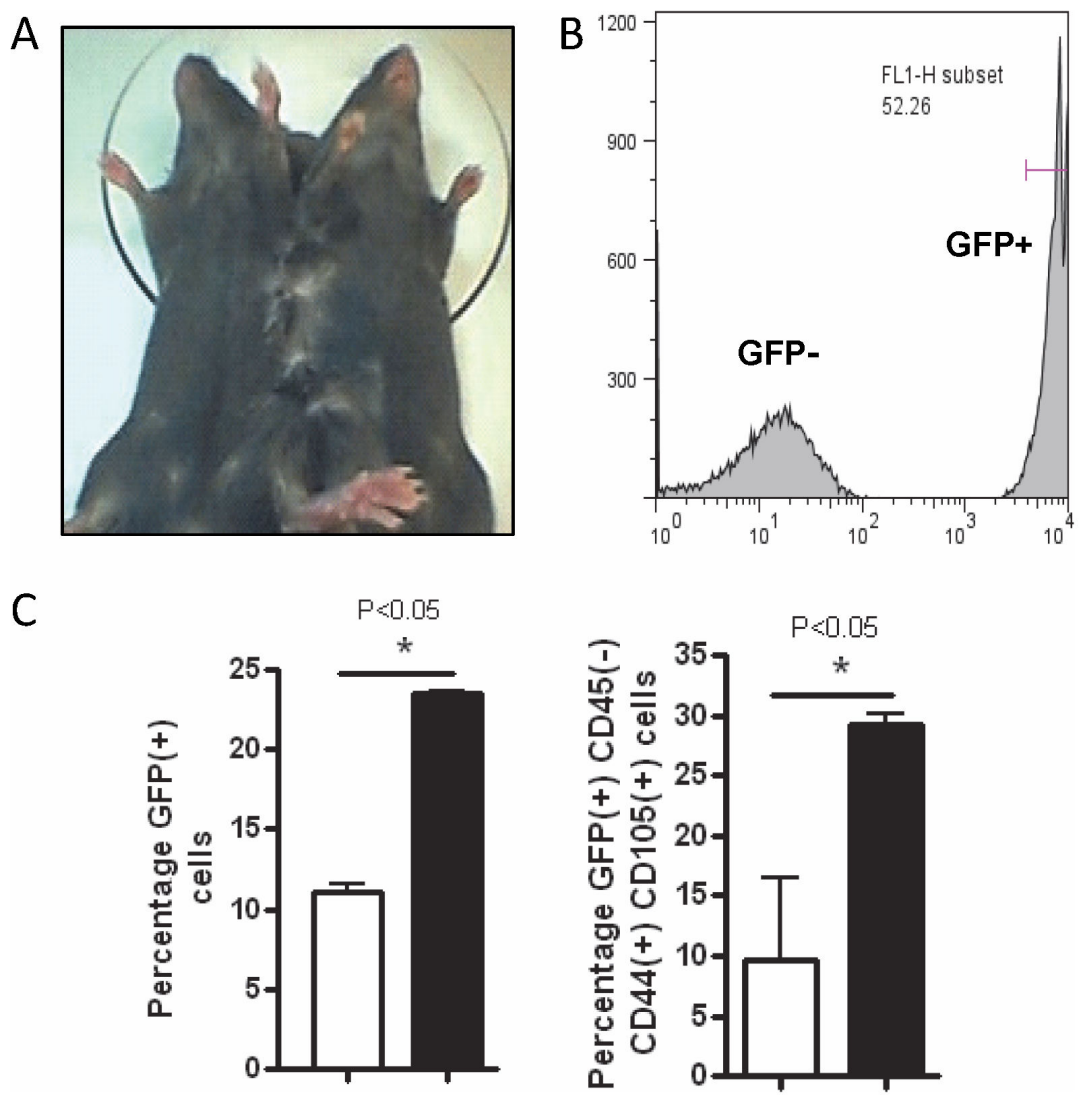
Migration of BMDMSCs to the bleomycin-injured lungs. *A*, Parabiont consisting of GFP-positive (GFP+) and wild-type (GFP-) mouse is shown. *B*, Flow cytometry analysis of leukocytes from the three sets of pooled blood of three parabionts (experiment using 9 parabiont pairs) showing roughly half of the cells show green fluorescence consistent with GFP positivity. *C*, Flow cytometry analysis of 200,000 cells prepared from three lungs of bleomycin- (black) or saline-treated (white) parabionts at day-21 are shown. Graphs compare percent of total cells that is GFP+(left) and percent of GFP+cells that is CD45-/CD44+/CD105+ (right). Error bars represent standard error of the mean (SEM).

**Figure 3:**
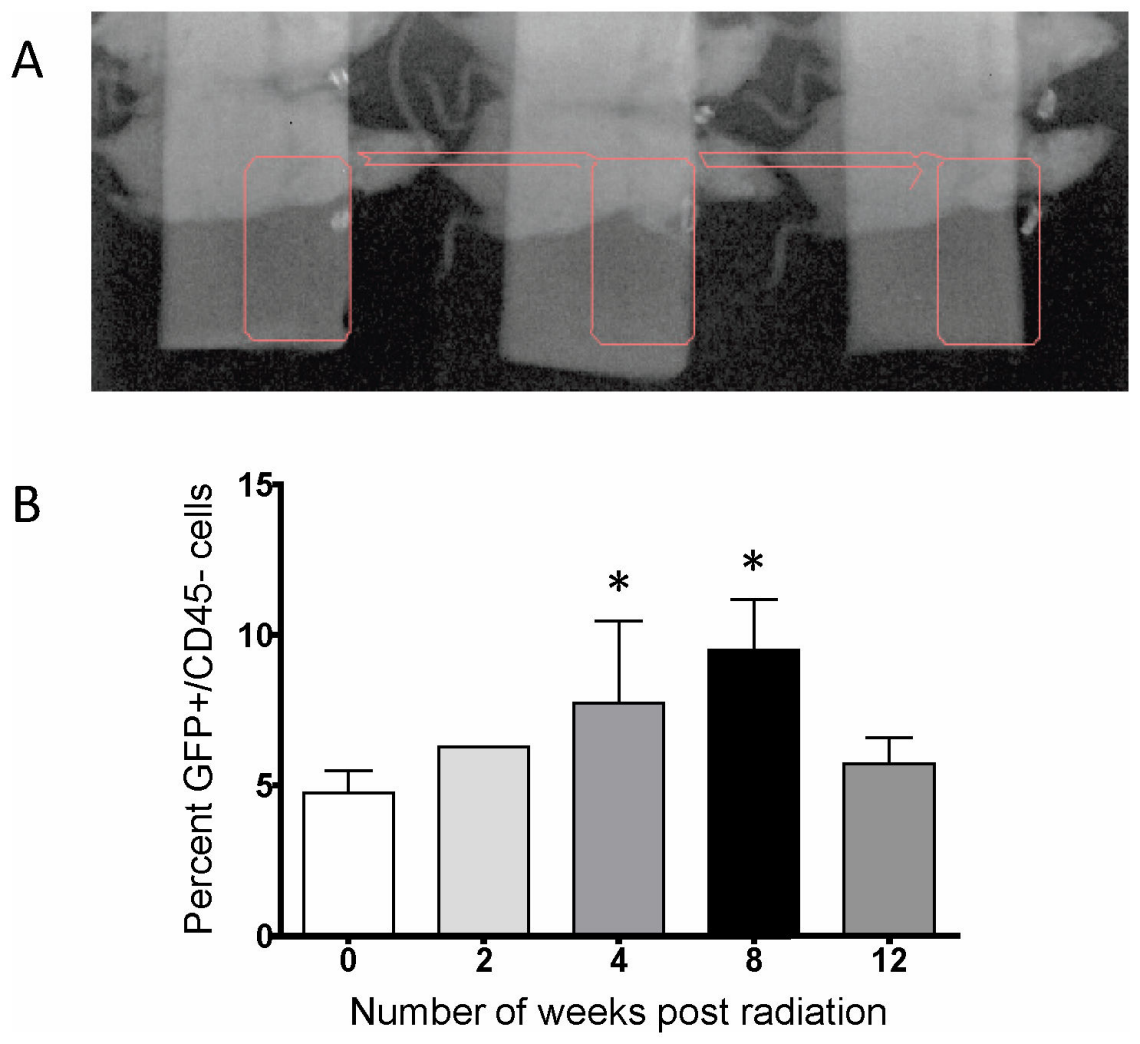
Use of parabiosis in hemithoracic irradiation animal model. *A*, Representative portal image of three parabionts receiving right hemithoracic radiation (region of treatment in pink) is shown. *B*, Graph (n=3) compares percent of GFP+/CD45-cells in the lungs at 0, 2, 4, 8, and 12-weeks post-irradiation. Error bars represent SEM. * indicates statistically significant difference (p<0.05) compared with value at 0 weeks.

A transgenic C57BL/6 mouse expressing GFP under the control of the β-actin promoter was surgically joined to a wild-type mouse to allow sharing of blood circulation (Figure **2A**) as previously reported [12]. Mice were housed together > two weeks before surgery to increase compatibility. Once anesthetized, elongated diamond-shaped skin pieces (long-axis extending from shoulder to hip, 1.0 cm short-axis width) were removed from opposite sides of each mouse. Exterior shoulder and hip muscles were sutured together with 4-0 prolene sutures and skin was joined with a continuous 3-0 prolene suture. GFP+ (left) and wild-type (right) mice make up the parabiotic pair. Cross-circulation is usually established by day 3 and completed by day 7. Once established (at least 2-4 weeks post-parabiosis), right hemithoracic irradiation is performed on the wild-type half of the parabiont (Figure **3A**). Irradiated lungs are harvested at various time-points for flow cytometry analysis to confirm presence of recruited BM-derived cells as previously described [5]. As a control, the wild-type half of parabiont was subjected to bleomycin-induced lung injury [5] and sacrificed at day-21. Lungs were again harvested for flow cytometry analysis. 

### BALF and serum CXCL12 protein concentration

**Figure 4:**
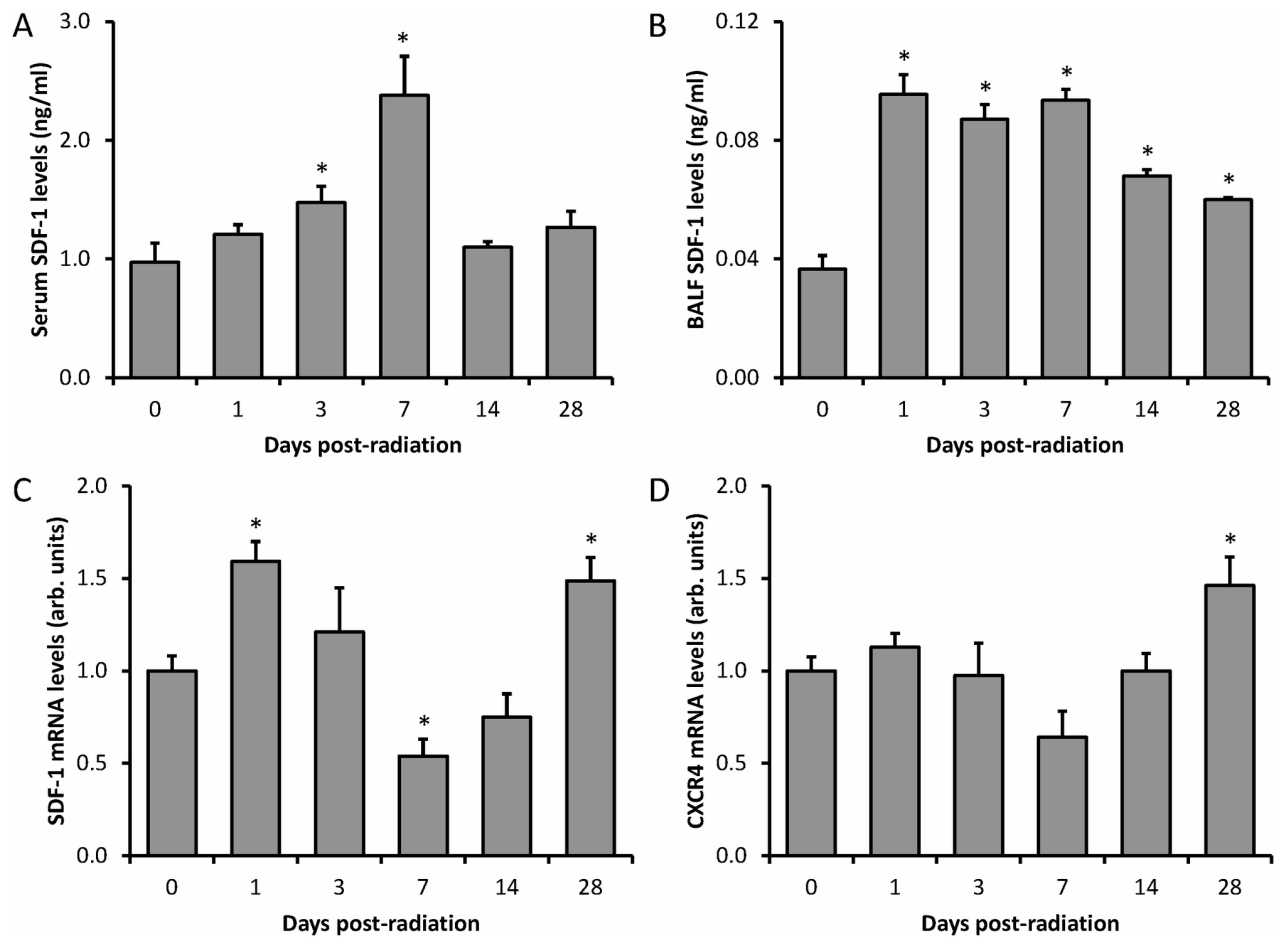
CXCL12 and CXCR4 expression in lungs of mice after 20 Gy radiation. Graphs of CXCL12 levels (ng/ml) (n=5 or 6) measured by ELISA in (*A*) serum and (*B*) BALF as well as (*C*) CXCL12 and (*D*) CXCR4 normalized mRNA levels (n=5) measured by RNase protection assay in lungs at 0, 1, 3, 7, 14 and 28-days post-irradiation (whole thorax) are shown. Error bars represent SEM. * indicates statistically significant difference (p<0.05) compared with value at day-0.

Mouse bronchoalveolar lavage fluid (BALF) and serum CXCL12 concentrations were measured at 0, 1, 3, 7, 14, and 28 days after whole thoracic irradiation. To obtain BALF, mice tracheae were cannulated with 20-guage needles, and lungs were instilled with 0.6-mL of PBS and recovered twice. The BALF was pooled and cells/debris spun down. Blood was obtained by heart puncture in sacrificed mice and cellular components were removed by centrifugation. The concentration of CXCL12 in the BALF and serum was determined by ELISA, according to manufacturer’s recommendations (R&D Systems). Results were graphed as absolute concentrations based on a standard curve developed for each experiment (Figure **4A-B**). 

### CXCR4/CXCL12 mRNA quantitation

Lung CXCR4 and CXCL12 mRNA levels were determined at the same time-points. Flash frozen lung samples were used for extraction of total RNA with Trizol (Invitrogen). mRNA quantitation was determined by RNase protection assay as previously described [13] except that the probes used for detection of CXCL12 and CXCR4 span 369 nucleotides (base 39-407 of mRNA) and 263 nucleotides (base 447-709 of mRNA), respectively. Protection of actin mRNA (Ambion) was used as the normalization control. Protected RNAs were detected by phosphor imaging on Typhoon 9210 using ImageQuant software from Molecular Dynamics (GE Healthcare). Results are graphed as relative levels with the day 0 values arbitrarily set at one based on a standard curve developed for each experiment (Figure **4A-B**). 

### Histology

**Figure 5:**
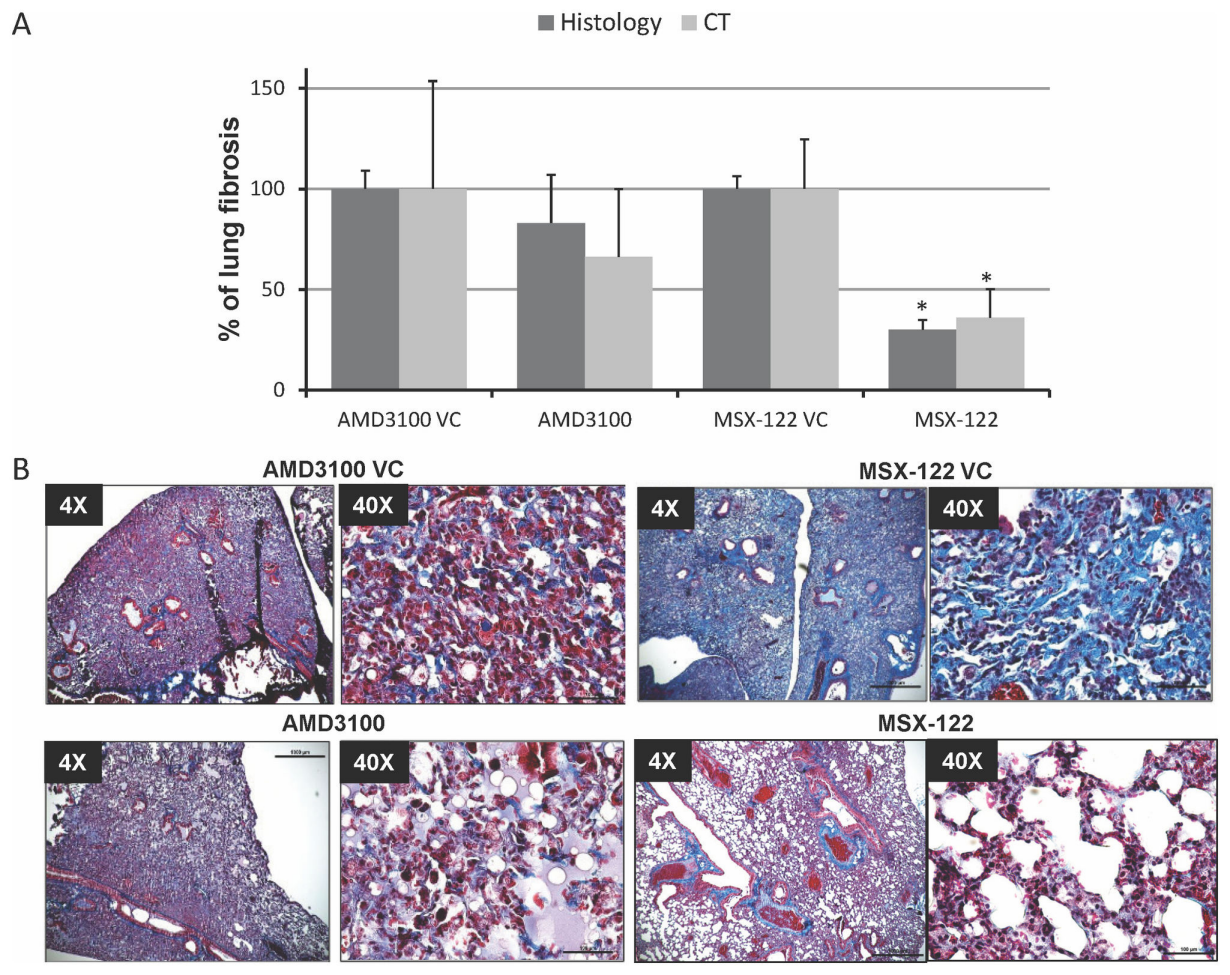
CXCR4 antagonists inhibit radiation-induced PF. Mice underwent hemithoracic irradiation (20-Gy) and treated with CXCR4 antagonists or their corresponding vehicle controls (n=10/group). *A*, Lungs were evaluated histologically after trichrome staining (dark bars) or by CT scan (light bars) at 20-weeks post-irradiation. Histologic evaluation was scored (0-12) in each lung as described in the Methods section. CT scan evaluation was scored as the percent volume of lung with density>-200 HU. In all assessments, the vehicle controls values were normalized to 100% with drug-treated values expressed relative to corresponding controls to evaluate the efficacy of the CXCR4 antagonists in attenuating radiation-induced PF. Error bar represents SEM. * indicates statistically significant difference (p<0.05) compared with vehicle controls. *B*, Representative micrographs of trichrome staining are shown in 4X and 40X magnification.

At 20-weeks post-irradiation, all lungs were collected and fixed with 4% paraformaldehyde for 24 hours, paraffin-embedded and sectioned at 6-μm. Slides were then shipped to the University of Pittsburgh where they were stained with hematoxylin and eosin (H&E) and Masson’s trichrome. A blind quantification was carried out in which the severity of the fibrosis (cell numbers and strength of staining) is quantified from 0 (normal) to IV severe (no airspace), II and III are intermediate. We selected 3 slides of lungs (middle, 50-µm above and below from the middle) per animal. Therefore the maximum PF score per animal is 12-point (100% in Figure **5A**). In addition to the 20 week-irradiated groups, mice in the different groups were sacrificed at earlier time points (at 0, 8, 12, and 16 post-irradiation, n=6 per group) for histology-based assessment of PF. 

### Administration of CXCR4 antagonists

Irradiated mice were subject to treatment with the CXCR4 antagonists AMD3100 and MSX-122 (n=10 each). Daily subcutaneous injections (s.c.) of 10 mg/kg AMD3100 in 100-μL vehicle (PBS) or intraperitoneal injections (i.p.) of 10 mg/kg MSX-122 in 100-μL vehicle (dissolved in 10% DMSO and 90% of 45% 2-hydroxypropyl-β-cyclodextrin (CD) (Fluka) in PBS) were given over 20 weeks (initiated 24 hours before irradiation). To assess the treatment outcome, AMD3100-treated mice were compared with subcutaneous PBS and MSX-122-treated mice were compared with i.p. 10% DMSO/45% CD vehicle controls. 

### Statistical Methods

For comparison between groups, unpaired, two-tailed student’s t-test was used. 

## Results

### CT-based fibrosis assessment correlates to histology in hemithoracic radiation-induced PF model

Whole-thoracic irradiation to mice results in significant morbidity and mortality. Therefore, we established a hemithoracic irradiation model that yielded less than 5% radiation-induced mortality (20 Gy, single dose) up to 20 weeks post-irradiation. CT scans were obtained on irradiated mice and the region of interest (whole lung) was contoured on each CT slice to define 3D-volumes of interest (Figure **1B**). The irradiated lungs of representative mice at the 0, 8, 12, 16, and 20 week time points were compared histologically and by CT scan. H&E sections revealed pathologic changes such as decreased alveolar spaces, thickened alveolar walls and increased inflammatory cells and fibroblasts, clearly indicating the radiation-induced lung injury and fibrosis over period of the time (Figure **1C**). The pathologic progression over time correlated well with representative axial CT (Figure **1D**), suggesting the increase in the degree of radiation-induced lung injury by plotting Hounsfield Units (HU) versus % volume of the irradiated lung (Figure **1E**). 

Based on preliminary data, an arbitrary HU of -200 was set as the threshold value for the presence of significant lung hardening (% volume at -200 HU means the percent of the lung whose tissue density is ≥ -200 HU, V_-200_). For the unirradiated lung, normal alveolar structure is seen and median HU (HU_50_) is ~ -500 while V_-200_ is < 5% (Fig. 1C-E.1). For the 12 week-irradiated lung, HU_50_ shifts to the right and V_-200_ remains < 10% (Fig. 1E.2); histologic evaluation of the same lung showed patched areas of interstitial widening due to infiltration of inflammatory cells and interstitial fibrosis (Fig. 1C.2 **and data not shown**). At 16 weeks, the H&E staining of the lung exhibits an increased interstitial thickening in association with hyperplasia of alveolar lining cells, loss of normal alveoli and dilatation of alveolar ducts and remaining alveoli with HU_50_ ~ -200 and V_-200_ ~ 50% (Fig. 1C-E.3). Finally, by 20 weeks, the entire irradiated lung has opacified on CT scan, consistent with finding of a solid lung in association with extensive infiltration of inflammatory cells and interstitial fibrosis (Fig. 1C-E.4). 

### Parabiont model demonstrates recruitment of mesenchymal stem cells

#### Bleomycin-treated parabiont

The origins of fibrosis-causing fibrocytes in idiopathic PF (lungs versus other sources) have been debated. Xu et al. showed in a bleomycin-induced PF mouse model that BM-derived CXCR4+ stem cells are recruited to the exposed lung due to CXCL12 expression in response to bleomycin [5]. We hypothesized that circulating CXCR4+ BM-derived mesenchymal stem cells (BMDMSCs) are recruited to irradiated-lungs and that this process is critical for ultimate development of PF. To provide stronger evidence for this idea, we adapted a parabiotic model where regenerating tissues in an injured animal could be exposed to marked circulating cells of a non-injured animal. This is accomplished by forcing development of vascular anastomoses between two syngeneic animals resulting in a single, shared circulatory system. Parabiont consisting of GFP-expressing transgenic mouse on the left and wild-type mouse on the right is shown (Figure **2A**). Flow analysis of leukocytes from the blood of this parabiont indicates that approximately half are GFP+ (Figure **2B**). In this model, at day 21 after treatment of the wild-type half with bleomycin, a significant increase in the proportion of GFP+ cells in the lungs is seen compared with untreated controls (Figure **2C** **, left**). We next sought to determine what percent of the detected GFP+ cells are mesenchymal stem cells. Surface proteins including CD44 and CD105 are generally expressed in BMDMSCs while CD45, a hematopoetic marker, is generally not expressed in these cells. The percent of GFP+ cells that are CD45-/CD44+/CD105+ in the bleomycin-treated lungs is significantly increased compared with untreated lungs (Figure **2C** **, right**). This experiment provides further evidence that bleomycin-induced lung injury results in the accumulation of CXCR4+ mesenchymal stem cells in the lung that are likely to be BMDMSCs although the contribution of other cellular compartments that may serve as a source of these cells can not be ruled out. 

#### Radiation-treated parabiont

To assess whether non-hematopoietic BM stem cells, rather than regional cells, are similarly recruited to the irradiated-lung, the right hemithorax of the parabiotic wild-type half was irradiated (Figure **3A**) and the irradiated lung was collected. Because only the right lung was collected per parabiont, there was insufficient yield of cells to perform triple staining for CD45/CD44/CD105. We therefore assessed for GFP+ cells that lacked expression of CD45 in the irradiated lung. Quantitation of GFP+/CD45- cells shows a trend of increasing recruitment of circulating stem cells that are of non-hematopoietic lineage out to 8-weeks post-irradiation (Figure **3B**). 

### Lung irradiation increases activity of the CXCR4/CXCL12-axis

We sought to determine whether the CXCR4/CXCL12-axis responds to lung irradiation by measuring the serum and BAL CXCL12 concentration, and CXCR4 and CXCL12 mRNA level in the lungs at 0, 1, 3, 7, 14, and 28 days post-whole thoracic irradiation. Both serum and BAL CXCL12 concentrations showed fluctuating but increasing trends over the 28-day course. In particular, BAL CXCL12 concentrations remained at consistently and significantly higher levels out to day-28 compared with the day-0 level (Figure **4A-B**). Lung CXCR4 and CXCL12 mRNA levels also fluctuated but showed a general trend of increase with the peak at day-28 post-irradiation (Figure **4C-D**). 

### MSX-122 attenuates radiation-induced PF

To test the impact of CXCR4 blockade with either AMD3100 or MSX-122 on development of radiation-induced PF, we administered AMD3100, 10 mg/kg, s.c. or MSX-122, 10 mg/kg i.p. for 20-weeks. We found from our initial pilot experiment that a single 20-Gy dose to the lung at the 20-week time-point resulted in fully 90% of untreated mice developing PF confirmed by both trichrome staining-positivity (maximum score 12) and CT scan showing a significant volume with density > -200 HU. Of note, AMD3100 was administered subcutaneously because of significant mortality with intravenous or intraperitoneal administration. Both histology-based and CT-based assessments show that MSX-122 significantly attenuated the development of fibrosis by 70%, while AMD3100 only trended towards reduction in fibrosis (Figure **5A**). Representative micrographs of trichrome-staining in each group at low and high power are shown, with obvious blue staining in the vehicle only group indicating significant deposition of collagen (Figure **5B**). The AMD3100-treated group showed 50% mortality during the 20-weeks of treatment, while no mortality was observed in the MSX-122- or vehicle-treated (irradiated) groups. 

## Discussion

Development of a therapy to prevent the development of PF could be of great benefit for at-risk patient populations. Because CXCR4/CXCL12 interaction is important for development of bleomycin-induced PF through recruitment of BM-derived progenitor cells [4,5,7], we investigated whether it is also critically involved in radiation-induced PF and whether blocking CXCR4 with our novel compound, MSX-122, can alleviate this process. 

In our assessment, volumetric CT image analysis correlated well with histological outcome. Our quantitative CT-based assessment of PF is novel because it involves whole-lung assessment, while typical histology analysis and scoring are limited to a few selective sections per lung. Previous efforts at characterizing PF with CT were similarly limited to the scoring of selective regions [14,15]. In the future, this methodology may serve as a non-invasive drug discovery tool for monitoring PF or relevant lung injury development in mice.

Blocking CXCR4 using an antagonist such as TN14003 or AMD3100 has been shown to effectively alleviate bleomycin-induced PF [5,7]. We found that MSX-122 is similarly effective [10,16]. Our current investigations also show that MSX-122 can alleviate radiation-induced PF. However, AMD3100 did not block this process to a significant extent in our system (Figure **5**). 

MSX-122 was discovered as one of the most potent CXCR4 inhibitors with reasonable bioavailability and has been carried forward as a clinical drug candidate. It displays high affinity binding to CXCR4 and inhibition of receptor function in the sub-nM range without metal-chelating capability [10]. The current clinical lead compound for CXCR4 inhibition, AMD3100, is a metal-chelating bicyclam and this chelating ability is believed to contribute to cardiotoxicity reported for this drug [17]. Metal-chelation may also account for increased mortality (50% @20-weeks) observed in mice treated with AMD3100. No such increased mortality was noted in mice treated with MSX-122 or the vehicle control groups. AMD3100 can also mobilize BM stem cells and was recently approved by the FDA for this purpose in patients undergoing harvesting of hematopoietic stem cells, which lacks in MSX-122. We speculate that this factor may contribute to increased effectiveness of MSX-122 compared with AMD3100 for blocking radiation-induced PF. While AMD3100 can decrease recruitment of CXCR4+ BMDMSCs to injured lung, it may also contribute to PF by increasing the availability of BMDMSCs in the peripheral circulation [18]. Therefore, while limited single use of AMD3100 is widely accepted, drugs with improved toxicity profiles may be required for long-term clinical use. 

Our results suggest that the CXCR4/CXCL12-axis is critical in the development of radiation-induced PF in a mouse model and that CXCR4 inhibition may alleviate potential radiation-induced lung injury. Examining possible use of MSX-122 for blocking PF among patients undergoing thoracic irradiation warrants further investigations. 
